# Differentiation of Bone Marrow Stem Cells in Presence of Human and Xenogeneic Acellular Dermal Matrix and Collagen Membrane: an *in vitro* Study

**DOI:** 10.30476/DENTJODS.2021.89497.1418

**Published:** 2022-06

**Authors:** Sepideh Gholamhoseinnia, Vahid Esfahanian, Shirin Amini Sedeh

**Affiliations:** 1 Postgraduate Student, Dept. of Periodontics, Faculty of Dentistry, Isfahan (Khorasgan) Branch, Islamic Azad University, Isfahan, Iran; 2 Dept. of Periodontics, Faculty of Dentistry, Isfahan (Khorasgan) Branch, Islamic Azad University, Isfahan, Iran

**Keywords:** Acellular Dermal Matrix, Regeneration, Bone, Collagen, Mesenchymal stem cells, Osteogenesis, Regeneration, Periodontal Guided Tissue

## Abstract

**Statement of the Problem:**

Predictable bone regeneration is an objective in implant and periodontal treatments and barrier membranes may play a significant role in osteogenic reconstruction and differentiation.

**Purpose:**

We compared the osteoblastic differentiation level of bone marrow stem cells in the vicinity of different barrier membranes.

**Materials and Method:**

In this experimental in vitro study, human collagen membrane (HCM; Regen), xenogeneic collagen membrane (XCM; Jason), human acellular dermal matrix (HADM; Regen), and xenogeneic acellular dermal matrix (XADM) were used in 4 groups. No membranes were used in the control group (5th group). Bone marrow stem cells with 150,000 cells/well density were added to the culture medium. Cellular differentiation was assessed through real-time quantitative reverse transcription polymerase chain reaction (QRT-PCR) for alkaline phosphatase (ALP) and osteopontin (OPN) gene expression, and Alizarin Red staining after 21 days. Data were analyzed using Kruskal- Wallis and Mann–Whitney statistical tests on SPSS 20 software (*p* Value< 0.05).

**Results:**

ALP gene expression was significantly higher in HCM group compared to other four groups (*p*< 0.009) followed by XADM, control, HADM and XCM groups, respectively (*p*< 0.001). OPN gene expression was significantly more prominent in HCM group compared to other groups (*p*< 0.01) followed by XADM group in which OPN gene was expressed significantly more than XCM group. OPN gene expression was not significantly different in HADM and control groups (*p*= 0.52). Light absorption rate was higher in HCM group compared with other groups (*p*< 0.012). Light absorption rate was not significantly different among HADM, XADM, and control groups (*p*> 0.05), though it was higher in XCM group (*p*= 0.009).

**Conclusion:**

Bone marrow stem cells show different levels of differentiation in the vicinity of different membranes. Generally, cell differentiation was more prominent in the vicinity of human collagen membrane.

## Introduction

Guided tissue regeneration, especially in the case of bone tissue, is an accepted technique for periodontal and maxillomandibular regeneration [ 1 ], implant insertion, and repairing peri-implant lesions [ 2 ] in order to regenerate bone and gingival tissues and restore the function and aesthetics [ 3 ].

The main objective of using barrier membranes in tissue regeneration process is to prevent the growth of the gingival corium and epithelium into the lesions and maintain space for regeneration [ 4 ]. An important feature of these membranes is their ability to improve adhesion, cellular proliferation, and differentiation. It has been proved that the properties of barrier membrane or framework such as its composition, surface roughness, and so on can affect cellular proliferation and differentiation in regenerative therapies, though it is not completely investigated [ 5 - 6 ].

Various synthetic and natural membranes are introduced with appropriate results [ 7 ], including absorbable collagen membrane [ 8 ]. Since alveolar bone and periodontal ligament contain collagen, collagen membranes may have additional advantages in regenerative therapies [ 9 ]. Acellular dermal matrix (ADM) is also used in periodontal surgeries. ADM as a connective tissue substitute is associated with preventing secondary palate surgeries, shorter surgery duration, less complications and patient discomfort, no limitation in the amount of donor tissue, possibility of multiple tooth treatment in one session, natural appearance, and better patient compliance. In addition, the manufacturers claim that it improves blood circulation and fibroblast accumulation [ 10 - 11 ]. Despite the mentioned advantages of ADM, there are worries about ethical issues due to its human origin, which leaded to introduction of xenogeneic acellular dermal matrix (XADM). Recently, a pig-derived ADM or mucoderm is introduced as an alternative to allograft or connective tissue graft. Its advantages include lower price, xenogeneic origin, and availability in high amounts. Several studies have proved the ability of XADM to improve *in vitro* proliferation of human fibroblast, osteoblast, and endothelial cells [ 12 - 14 ].

An important prerequisite in regenerative and tissue-engineering therapies is the presence of progenitor cells in the area and their proliferation and differentiation ability [ 15 - 16 ]. The mesenchymal bone marrow cells may differentiate to alveolar bone and periodontal ligaments [ 17 ]. Bone marrow-derived mesenchymal stem cells secrete the extracellular matrix, which is vital to osteogenic differentiation, and the mineralization of this matrix shows the terminal phase of osteoblast differentiation [ 18 ]. Thus, bone marrow stem cells are important in the periodontal regeneration and especially osteogenic augmentation.

Basudan *et al.* [ 19 ] compared the effects of absorbable collagen membrane and mucoderm in the osteogenic guided reconstruction in cranial lesions of rats. They found that the highest formation of new bone was in absorbable collagen+ xenograft and the lowest formation of new bone was in the mucoderm group. An *et al.* [ 20 ] concluded that pig-dermal collagen membrane might be used as a reliable membrane in the regeneration process. Pappalardo *et al.* [ 21 ] purposed ADM as a perfect membrane.

Since no comparative study have been conducted on effect of these barrier membranes on the level of osteoblastic differentiation in bone marrow stem cells, while histologic comparison is one of the most valuable research methods, this study intended to evaluate the abovementioned effect to provide a guide for choosing the proper membrane.

## Materials and Method

This experimental *in vitro* study was performed on bone marrow stem cells provided by Tehran Jahade-Daneshghahi Genetic Resource Center.

Firstly, bone marrow stem cells were transferred to enriched culture medium with 10% fetal bovine serum (FBS) and 2 millimole L-Glutamine and incubated at 37˚C, 95% air-5% carbon dioxide. Then, the cultured bone marrow stem cells were transferred to a bigger plate for further proliferation. For this purpose, namely cellular passage, cells were washed twice with phosphate buffer saline (PBS) and adequate amount of Trypsin was added. After reassuring cell isolation, serum-containing culture medium was added so that Trypsin effect gets neutralized with the inhibitory effect of some substances in the serum on the protease enzyme. Then, the cells were transferred to centrifuge system.

In the next step, four types of barrier membranes including human collagen membrane (HCM) (Regen, Itb, Tehran, Iran), xenogeneic collagen membrane (XCM) (Botiss dental Jason, Berlin, Germany), human acellular dermal matrix (HADM) (Regen, Itb, Tehran, Iran), and xenogeneic acellular dermal matrix (XADM) (Botiss dental Mucoderm, Berlin, Germany) were selected to assess their effects on the differentiation of bone marrow stem cells. Then, 1×1mm of each membrane was trimmed and put in a well. Then, based on the group, samples were kept for 21 days during which the medium was changed every other day to maintain cellular nourishment. The study and control groups were provided as (1) bone marrow stem cells+ differential medium for 21 days (control group), (2) bone marrow stem cells+ HCM+ differential medium for 21 days, (3) bone marrow stem cells+ XADM+ differential medium for 21 days, (4) bone marrow stem cells+ HADM+ differential medium for 21 days, and (5) bone marrow stem cells+ XCM+ differential medium for 21 days.

Alizarin Red (AR) staining was used to assess level of osteoblastic differentiation, by comparing their staining (red) via light microscope and light absorption at 450 nm wavelength by Elisa reader. These numbers define amount of calcium deposits due to osteoblastic differentiation [ 18 ]. Afterwards, quantitative reverse transcription polymerase chain reaction (QRT-PCR) was used to assess osteoblastic differentiation. In this test, expression of mRNA of genes related to osteoblastic differentiation was assessed in test and control groups in the course of time.

In this study, glyceraldehyde 3-phosphate dehydrogenase (GAPDH), osteopontin (OPN), and alkaline phosphatase (ALP) genes were assessed. ALP and OPN gene expression is related to osteoblastic differentiation, which respectively increases at the beginning and at the end of differentiation. GAPDH gene is a hosting gene, which is expressed in all cells at a relatively constant level that is used to compare the expression of other genes. On the other hand, TRIzol (Invitrogen Corp., California, USA) was added to some cells in control group. Then, RNA was extracted and cDNA was synthesized to assess the expression of the mentioned genes by QRT-PCR so that the level of gene expression in day 1 before differentiation was measured.

Collected data were analyzed with Kruskal-Wallis and Mann-Whitney tests in SPSS-20 software. *p* Value< 0.05 was considered significant.

## Results

Expression of GAPDH gene has shown that ALP and OPN genes were expressed. QRT-PCR revealed a significant difference in osteoblastic cell differentiation between ALP gene expression (*p*= 0.009) and OPN gene expression (*p*= 0.01) among the five study groups (Tables 1 and 2). Paired comparison of groups showed that ALP gene expression was significantly higher in HCM group compared to other groups (*p*< 0.001), followed by XADM which was significantly superior to control, HADM and XCM groups (*p*< 0.001). Gene expression in control group was higher than HADM and XCM groups (*p*< 0.001). Moreover, gene expression was significantly higher in HADM group compared to XCM (*p*< 0.001).

**Table 1:** The mean expression of alkaline phosphatase (ALP) gene after 21 days in five groups

Group	Mean±SD	*p* Value
Control	35.22±0.37 ^d,g,h,i^	0/009
HCM^1^	37.65±0.08 ^a,b,c,d^	
XADM^2^	35.92±0.03 ^a,e,f,g^	
HADM^3^	30.97±0.01 ^b,e,h,j^	
XCM^4^	29.47±0.24 ^c,f,i,j^	

a,b,c,d,e,f,g,h,i: a significant difference between the groups

1. Human Collagen Membrane

2. Xenogeneic Acellular Dermal Matrix

3. Human Acellular Dermal Matrix

4. Xenogeneic Collagen Membrane

**Table 2:** The mean expression of osteopontin (OPN) gene after 21 days in five groups

Group	Mean ± SD	*p* Value
Control	27.56 ± 0.12 ^d,g,i^	0.01
HCM^1^	35.35 ± 0.25 ^a,b,c,d^	
XADM^2^	30.77 ± 0.39 ^a,e,f,g^	
HADM^3^	27.83 ± 0.16 ^b,e,h^	
XCM^4^	29.51 ± 0.08 ^c,f,h,i^	

a,b,c,d,e,f,g,h,i: a significant difference between the groups

1. Human Collagen Membrane

2. Xenogeneic Acellular Dermal Matrix

3. Human Acellular Dermal Matrix

4. Xenogeneic Collagen Membrane

Paired comparison of groups showed that OPN gene expression was significantly higher in HCM group compared to other groups (*p*< 0.001), followed by XA-DM which was significantly superior to control, HADM and XCM groups (*p*< 0.039). Gene expression in XCM group was higher than HADM and control groups (*p*< 0.047). However, no significant difference was observed between HADM and control groups (*p*= 0.52).

Osteoblastic cell differentiation comparison was performed using AR staining which showed significant difference between five study groups in terms of light absorption (*p*= 0.012) (Table 3).

**Table 3:** The mean light absorption rate after 21 days in five groups

Group	Mean ± SD	*p* Value
Control	1.08 ± 0.12 ^d,g^	0.012
HCM^1^	2.26 ± 0.20 ^a,b,c,d^	
XADM^2^	1.33 ± 0.25 ^a,e^	
HADM^3^	1.50 ± 0.04 ^c,f^	
XCM^4^	0.84 ± 0.03 ^b,e,f,g^	

a,b,c,d,e,f,g: a significant difference between the groups

Human Collagen Membrane

Xenogeneic Acellular Dermal Matrix

Human Acellular Dermal Matrix

Xenogeneic Collagen Membrane

Paired comparison showed that light absorption was significantly higher in HCM group compared to the other four groups (*p*< 0.001). Light absorption was not significantly different among HADM, XADM and control groups (*p*>0.05) and light absorption of these groups was significantly higher than XCM (*p*< 0.009).

## Discussion

Stem cell differentiation to osteogenic osteoblast is a key part of regenerative and osteogenic augmentation therapies; thus, the level of osteoblastic differentiation of stem cells in the vicinity of four different types of barrier membranes was assessed in this study.

Duration is an important factor in the differentiation process. Often, 21 days is required for osteoblast differentiation in laboratory studies [ 22 - 23 ].

Osteoblastic differentiation and osteogenesis process consists of several steps. The first step is the expression of ALP and OPN genes. Then, these genes will lead to protein production. Afterwards, bone cells should get mature to secrete extracellular matrix. Finally, the most important function of a bone cell is its ability to secrete extracellular matrix and calcium deposit [ 18 ].

In order to evaluate the cell differentiation process, it is crucial to pay attention to the gene expression patterns in different steps. GAPDH gene is a hosting gene, which is expressed in all cells at a relatively constant level. This gene is used to compare the expression of other genes. This gene reveals the expression of ALP and OPN genes. ALP gene expression is the specific inducing marker of osteogenesis in the initial steps of differentiation. On the other hand, OPN gene expression defines the advancement of differentiation process [ 18 ]. So the role of each marker should be noticed.

The results have shown that level of differentiation based on ALP gene expression was highest in HCM group, followed respectively by XADM, control, HADM, and XCM groups. It shows that cell differentiation starts more rapidly in the vicinity of HCM.

Since the XCM used in this study (Jason) contained a high amount of type III collagen based on manufacturer claim [ 24 ], it may not bring the advantages of HCM containing type I collagen. This may be a probable factor of superiority of HCM over XCM.

The results in study groups indicated that differentiation level based on OPN gene expression was similar to ALP gene expression. OPN gene expression was higher in the vicinity of HCM followed by mucoderm, which shows the role of these membranes on gene expression during differentiation process.

In this study, collagen membrane and ADM were evaluated due to their high consumption and extended use in periodontal treatments. Moreover, XADM was also assessed because of its novelty and limited related studies. 

It is well proved that cellular adhesion, proliferation, and differentiation are affected by membrane properties, though it is not completely investigated [ 5 - 6 , 23 ]. According to Pabst *et al.* [ 23 ] study, the surface porosity of mucograft membrane and subsequently cellular adhesion is higher than (peg-derived) XCM, which may probably improve the osteoblastic differentiation of mucograft compared to XCM. Since OPN gene indicated the advancement of differentiation process, it can be concluded that XCM is superior to control group in the course of differentiation process advancement. Presence of collagen may be a probable reason of why the differentiation process occurs more rapidly.

Comparison of light absorbance in studied groups showed that the differentiation level was highest in HCM group in terms of calcium deposit and light absorbance, which shows better performance of osteoblastic differentiation in the vicinity of HCM. HADM, XADM, control, and XCM groups had the next levels, respectively. The results showed that the differentiation process could not reach final steps in the vicinity of XCM.

Considering ALP gene expression as the initiation of differentiation process, OPN gene expression as progression of differentiation process and AR test (measuring calcium deposit) as termination of differentiation process, results of this study indicates that differentiation process initiates, progresses, and terminates in the vicinity of HCM more rapidly compared to other groups. Stem cells acted well in initiation and progression of differentiation in the vicinity of XADM, though failed to terminate it as successfully. The results of XCM were heterogeneous and did not follow a specific pattern. Higher sample size may help reach more reasonable results.

No analogous studies were found evaluating and comparing these membranes, though there were some similar studies. For instance, Basudan *et al.* [ 19 ] evaluated the effect of absorbable collagen membrane and mucograft on guided bone regeneration in rats with cranial lesions. Best osteogenesis was observed after 8 weeks in absorbable collagen membrane+xenograft group. No significant difference was reported between mucograft+xenograft and absorbable collagen membrane groups. The least bone formation was observed in mucograft group. The superiority of HCM compared to mucograft in osteogenesis observed in their study was consistent with the result of current study.

Comparative evaluation of osteoblast-like cells adhesion and differentiation in the vicinity of HADM and tetrafluoroethylene in Liu *et al.* [ 25 ] study revealed no significant difference. Rothamel *et al.* [ 26 ] study on the effects of different collagen membranes on fibroblast and osteoblast adhesion and proliferation showed that bioguide, esix, and totodent membranes improved the fibroblast and osteoblast adhesion and proliferation, while biomand membrane prevented it.

Papaioannou *et al.* [ 27 ] evaluated the effect of five absorbable collagen membranes on osteoblast-like cells (MG63 cell line) adhesion and proliferation and concluded that the type of membrane affects cellular adhesion and proliferation.

Miron *et al.* [ 28 ] studied the adhesion and differentiation of osteoblasts in the vicinity of collagen membrane combined with a new type of enamel matrix derivatives. They reported increased expression of ALP gene, osteogenic sialoprotein gene, and AR staining in comparison to the control group. In another study, Miron *et al.* [ 29 ] assessed the proliferation and differentiation of human osteoblasts in the vicinity of collagen membranes combined with bone morphogenic protein 2 (BMP2) and transforming growth factor beta1 (TGFβ1). Adherence of osteoblasts to all these membranes and improvement of osteoblasts’ proliferation parameters compared to control group was observed. In addition, PCR analysis showed that BMP2 increased osteoblast differentiation markers. Moreover, based on AR staining, BMP2 leaded to improved mineralization of primary osteoblasts compared to control and TGFβ1 groups.

Kobayashi *et al.* [ 30 ] investigated the role of collagen membrane (bioguide) coated with bone conditioned media (extracted from cortical bone of pig mandible) on adhesion and differentiation of osteoblasts via real-time PCR and AR staining. They reported increased expression of alkaline phosphatase, osteocalcin, osteogenic sialoprotein, and increased AR staining intensity.

## Conclusion

The results of the current study suggest different behavior of bone marrow stem cells in osteoblastic differentiation process in the vicinity of different membranes. Osteoblastic differentiation and ontogenesis processes occurred significantly better in the presence of HCM compared to other membranes.

## Conflict of Interest

The authors declare that they have no conflict of interest.
